# Supplementary figures and images for: Homeostatic scaling is driven by a translation-dependent degradation axis that recruits miRISC remodeling
Source: PLoS Biol. 2021 Nov 23;19(11):e3001432. doi: 10.1371/journal.pbio.3001432 (PMC8610276; doi:10.1371/journal.pbio.3001432)

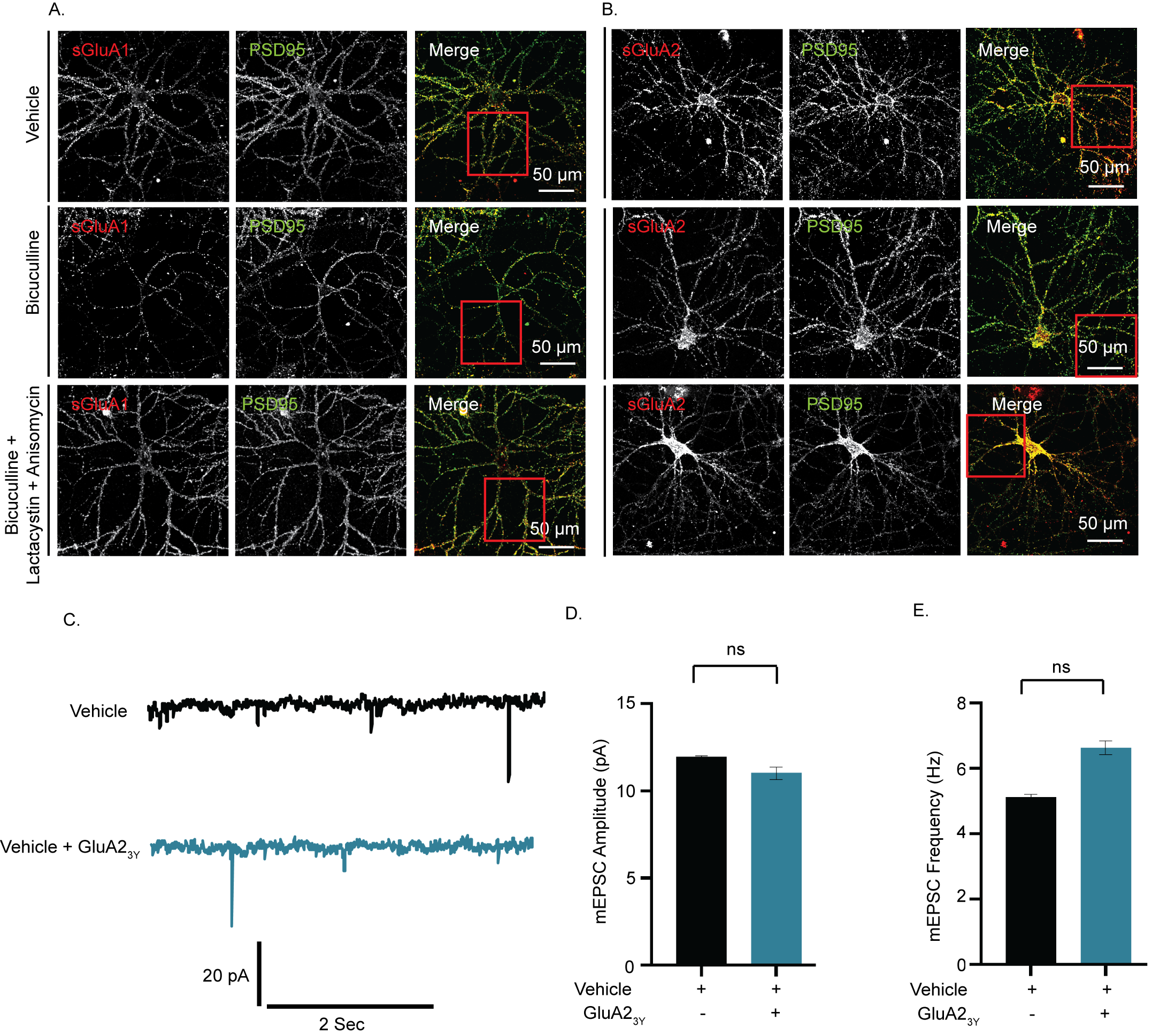

Supplement: S1 Fig — (A, B) Hippocampal neurons were stained for sGluA1 (A) or sGluA2 (B) and PSD95 as described in Fig 2A and 2B. Photomicrograph showing images for sGluA1 or sGluA2 (red) and PSD95 (green) and sGluA1/PSD95 or sGluA2/PSD95 (merged). High-magnification images of dendrites shown in Fig 2 marked in red square. Scale bar as indicated. Quantitation shown in Fig 2C and 2D. See Fig 2 for data. (C-E) mEPSCs traces from hippocampal neurons (DIV 18–24) treated with vehicle or GluA23y for 24 hours (C) as described in Fig 2E. Scale as indicated. Mean mEPSC amplitudes (D) and frequencies (E) in neurons treated as indicated. n = 12. Data shown as mean ± SEM. One-way ANOVA and Fisher’s LSD. See Fig 2 for data. The data underlying this figure are available at https://figshare.com/articles/dataset/Homeostatic_scaling_is_driven_by_a_translation-dependent_degradation_axis_that_recruits_miRISC_remodeling/16768816. AMPAR, AMPA receptor; DIV, days in vitro; mEPSC, miniature excitatory postsynaptic current; ns, not significant. (TIF) [file pbio.3001432.s001.tif]

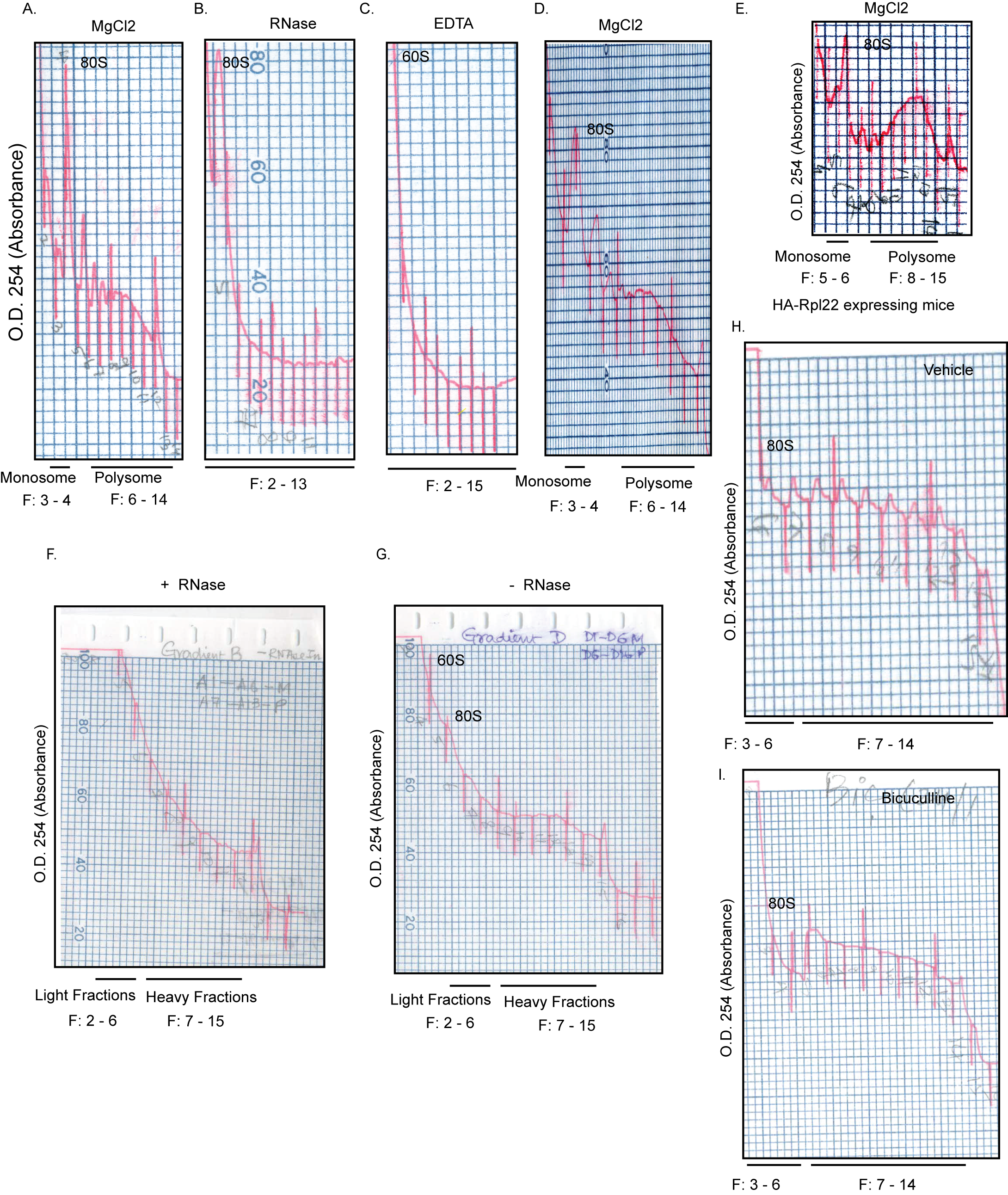

Supplement: S2 Fig — (A-G) A254 profile obtained from spectrophotometer attached to gradient fractionator shown in Figs 3 and 4. Traces were drawn from original A254 profile obtained from hippocampal cytoplasmic extract treated with MgCl2 (A), RNase (B), EDTA (C), and MgCl2 (D) shown in Fig 3, MgCl2-treated extract from mouse expressing HA-Rpl22 in excitatory neurons from hippocampus (E) shown in Fig 4C and RNase (F) or without RNase (G)–treated extract from mouse expressing HA-Rpl22 in excitatory neurons from hippocampus shown in Fig 4J. (H, I) A254 profile of sucrose density fractions obtained from vehicle (H) or bicuculline (I)–treated cortical neurons. Traces were drawn from these original A254 profiles as shown in Fig 5A and 5B. (TIF) [file pbio.3001432.s002.tif]

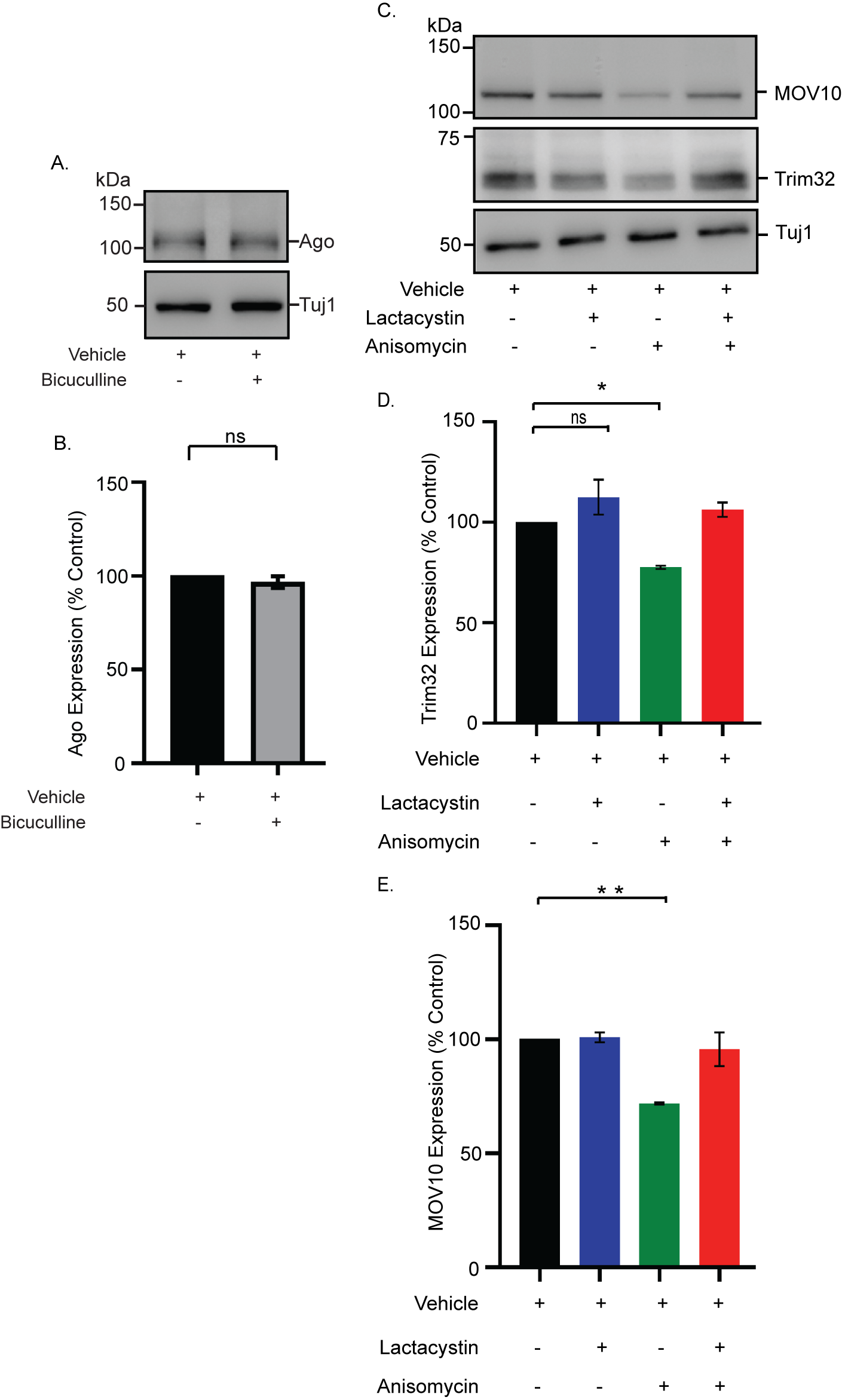

Supplement: S3 Fig — Hippocampal neurons (DIV 21) were treated with bicuculline for 24 hours. (A) Photomicrograph showing the expression of Ago and Tuj1 as detected by western blot analysis. (B) Quantitation of Ago expression. n = 3. ns, not significant. Unpaired 2-tailed t test with Welch’s correction. (C-E) Hippocampal neurons (DIV 21) treated with lactacystin, anisomycin, and both for 24 hours. Photomicrograph showing the expression of Trim32 and MOV10 as detected by western blot analysis (C). Quantitation of Trim32 (D) and MOV10 (E). Data shown as mean ± SEM, n = 3, *p < 0.001 and **p < 0.0003. One-way ANOVA and Fisher’s LSD. See Fig 6 for data. The data underlying this figure are available at https://figshare.com/articles/dataset/Homeostatic_scaling_is_driven_by_a_translation-dependent_degradation_axis_that_recruits_miRISC_remodeling/16768816. Ago, Argonaute; DIV, days in vitro; miRISC, miRNA-induced silencing complex; ns, not significant. (TIF) [file pbio.3001432.s003.tif]

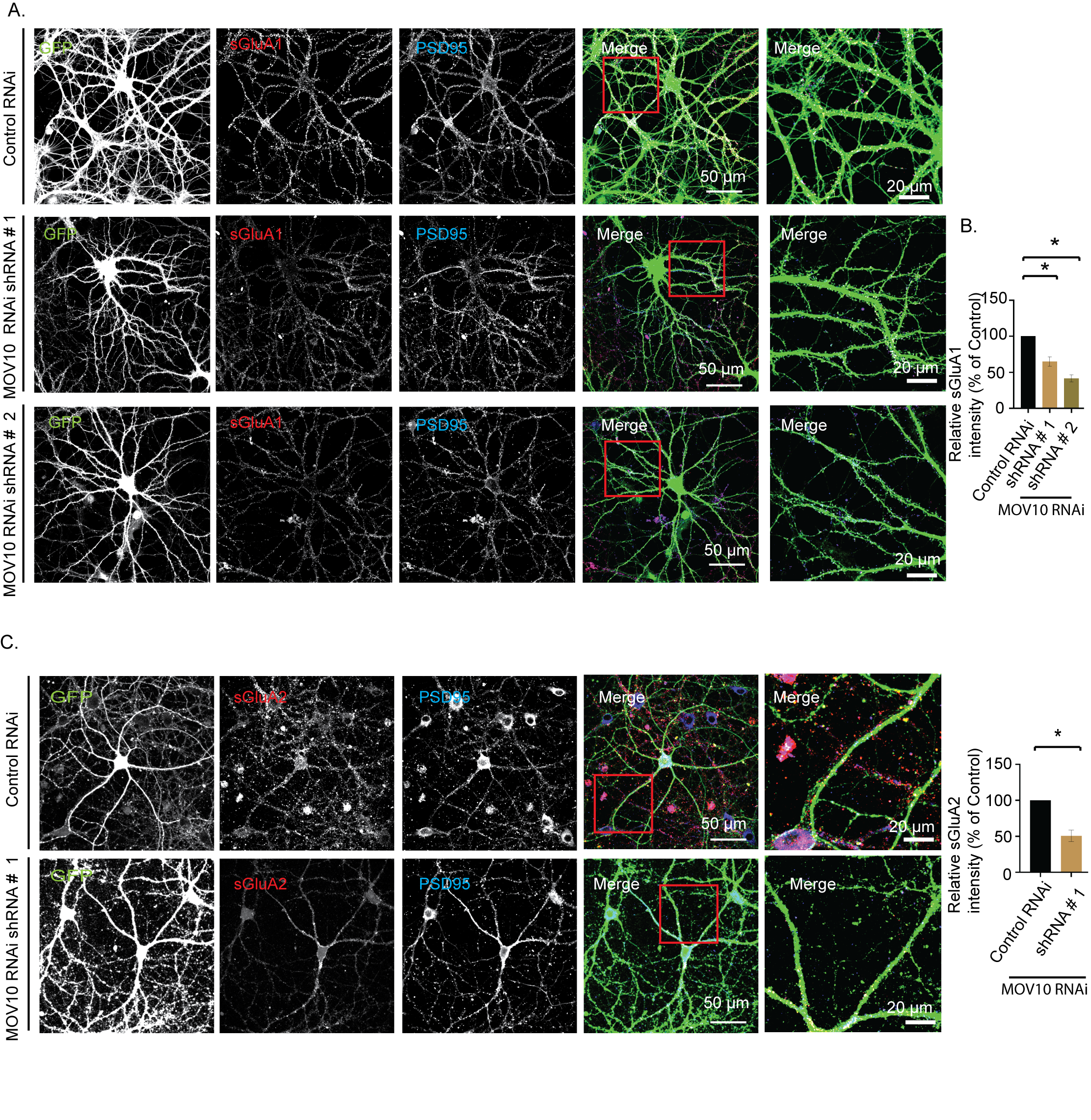

Supplement: S4 Fig — Hippocampal neurons (DIV 14–15) transduced with lentivirus expressing two shRNAs against MOV10 (shRNA#1 or shRNA#2) along with GFP. Transduced neurons (DIV 21–24) were immunostained for surface GluA1 (sGluA1) and coimmunostained for PSD95. (A) Photomicrograph showing confocal images of GFP (green), sGluA1 (red), PSD95 (blue), and GFP/sGluA1/PSD95 (merged). High-magnification images of dendrites shown in Fig 9 marked in red square. (B) Relative intensity of sGluA1 particles at the synapse (overlap with PSD95 particles onto GFP expressing dendrites). Normalized intensity of sGluA1 relative to control was plotted. Data shown as mean ± SEM. *p < 0.01. One-way ANOVA and Fisher’s LSD. (C) Hippocampal neurons (DIV 14–15) transduced with lentivirus expressing shRNA against MOV10 (shRNA#1) along with GFP. Transduced neurons (DIV 21–24) were immunostained for surface GluA2 (sGluA2) and PSD95. Photomicrograph showing confocal images of GFP (green), sGluA2 (red), PSD95 (blue), and GFP/sGluA2/PSD95 (merged). High-magnification images of dendrites shown in Fig 9 marked in red square. Scale as indicated. Relative intensity of sGluA2 particles at the synapse (overlap with PSD95 particles onto GFP expressing dendrites). Normalized intensity of sGluA2 relative to control was plotted. Data shown as mean ± SEM. *p < 0.01. One-way ANOVA and Fisher’s LSD. See Fig 9 for data. The data underlying this figure are available at https://figshare.com/articles/dataset/Homeostatic_scaling_is_driven_by_a_translation-dependent_degradation_axis_that_recruits_miRISC_remodeling/16768816. AMPAR, AMPA receptor; DIV, days in vitro; sAMPAR, surface AMPAR. (TIF) [file pbio.3001432.s004.tif]

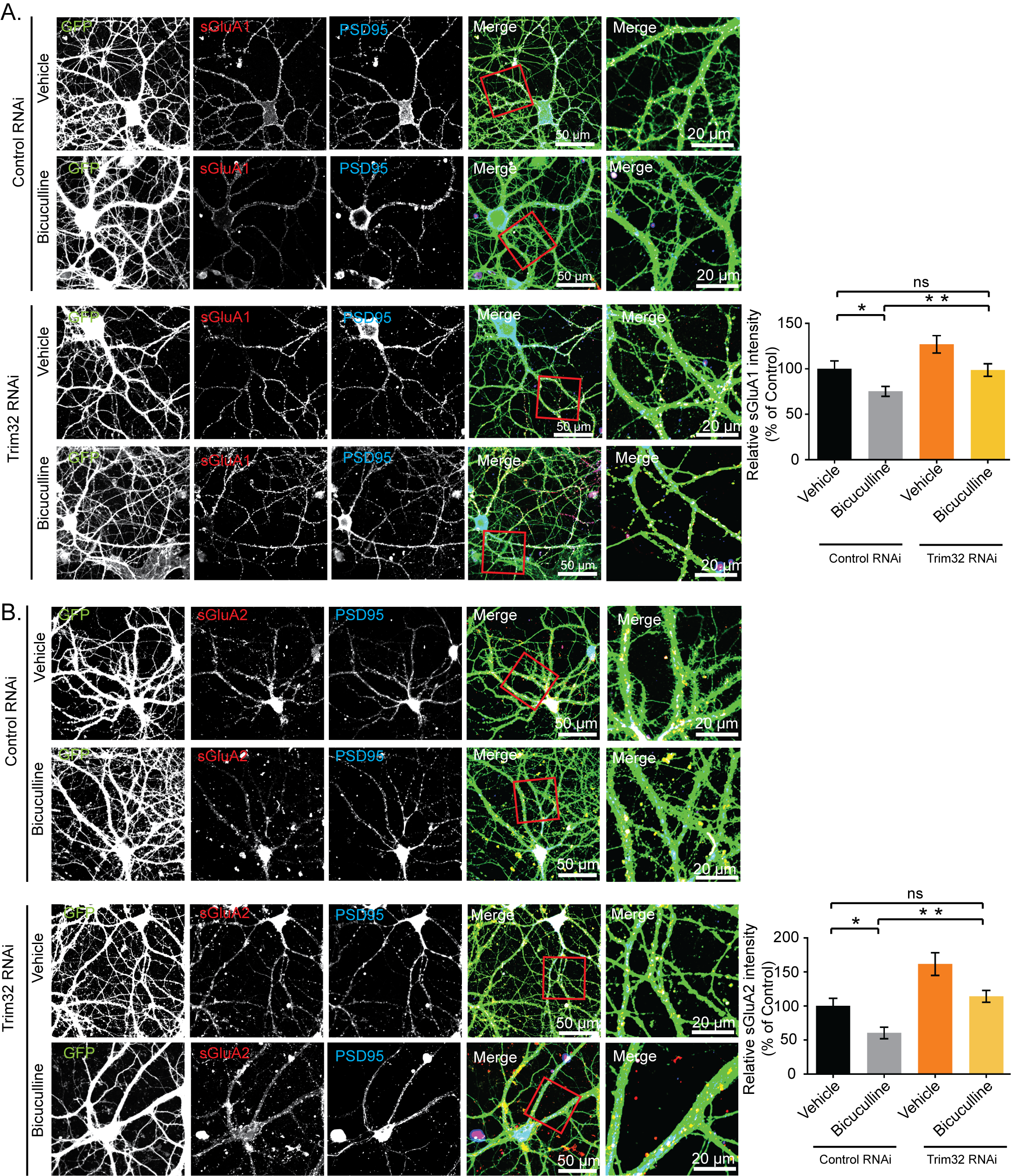

Supplement: S5 Fig — Hippocampal neurons (DIV 14–15) transduced with lentivirus expressing shRNA against Trim32 along with GFP. (A-B) Transduced neurons (DIV 21–24) were stimulated with bicuculline for 24 hours and immunostained for sGluA1 (A) or sGluA2 (B) and coimmunostained for PSD95. Photomicrograph showing confocal images of GFP (green), sGluA1/sGluA2 (red), PSD95 (blue), and GFP/sGluA1 or sGluA2/PSD95 (merged). High-magnification images of dendrites shown in Fig 12 A-F marked in red square. Relative intensity of surface GluA1 (A) or surface GluA2 (B) particles at the synapse (overlap with PSD95 particles onto GFP expressing dendrites). Normalized intensity of surface GluA1/GluA2 relative to control was plotted. Data shown as mean ± SEM. *p < 0.02, **p < 0.03, for sGluA1. *p < 0.008, **p < 0.002, for sGluA2. One-way ANOVA and Fisher’s LSD. See Fig 12 for data. The data underlying this figure are available at https://figshare.com/articles/dataset/Homeostatic_scaling_is_driven_by_a_translation-dependent_degradation_axis_that_recruits_miRISC_remodeling/16768816. AMPAR, AMPA receptor; DIV, days in vitro; ns, not significant; sAMPAR, surface AMPAR. (TIF) [file pbio.3001432.s005.tif]

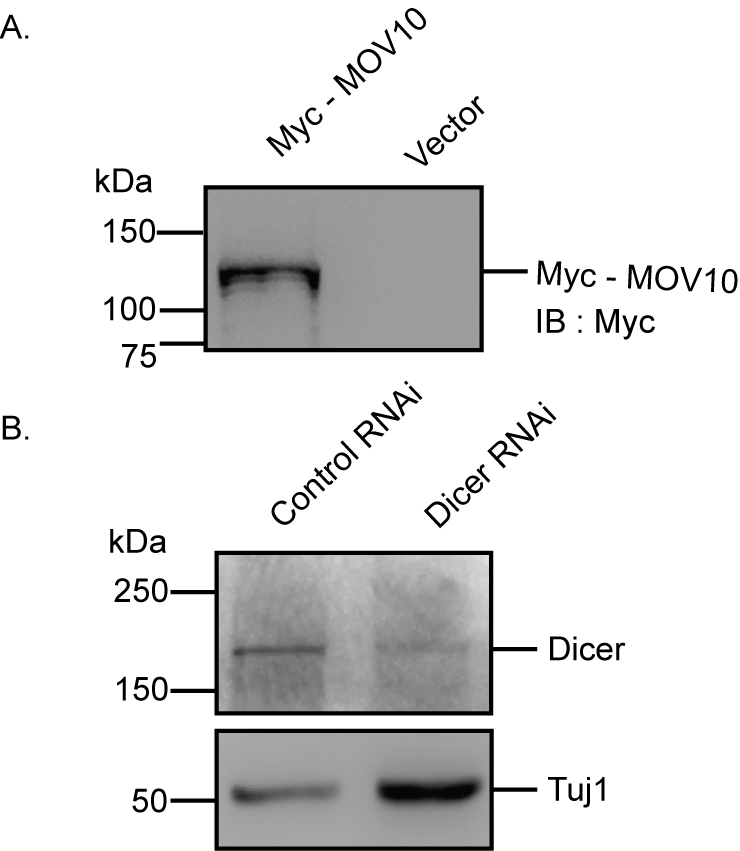

Supplement: S6 Fig — (A) Myc-tagged MOV10 was transfected in hippocampal neurons (DIV 15) as detected by western blot analysis (DIV 21) using antibody against Myc. See also Fig 10. (B) Hippocampal neurons (DIV 14) were transduced with lentivirus expressing shRNA against Dicer or control shRNA. Photomicrograph showing effective knockdown of Dicer (DIV 24) as detected by western blot analysis using antibody against Dicer. See data for Fig 13. The data underlying this figure are available at https://figshare.com/articles/dataset/Homeostatic_scaling_is_driven_by_a_translation-dependent_degradation_axis_that_recruits_miRISC_remodeling/16768816. DIV, days in vitro; IB, immunoblot. (TIF) [file pbio.3001432.s006.tif]
