# Supplementary material for: Homeostatic scaling is driven by a translation-dependent degradation axis that recruits miRISC remodeling
Source: PLoS Biol. 2021 Nov 23;19(11):e3001432. doi: 10.1371/journal.pbio.3001432 (PMC8610276; doi:10.1371/journal.pbio.3001432)
Supplement: Attachment — Submitted filename: CTD rebuttal letter FINAL July 20th 2021.pdf [file pbio.3001432.s009.pdf]

Dear Dr. Smith,

We thank you for the reviews on our manuscript “Homeostatic scaling is driven by a translation-dependent degradation axis that recruits miRISC remodeling” (manuscript # PBIOLOGY-D-20-02451R1). We welcome your advice for submitting the revised version as a new submission after significant addition of data to bolster our proposed model. Briefly, the new evidences favoring our claim are as follows.

1) Genetic manipulations of Trim32 (Trim32 RNAi) and MOV10 (myc-MOV overexpression) leads to a partial impairment of downscaling, which is opposite to the pattern of downscaling observed due to bicuculline mediated *de novo* Trim32 translation and MOV10 degradation.

2) Loss of Trim32 prevents chronic hyperactivity-induced reduction in sAMPARs that is commensurate with an increase in mEPSC amplitude.

3) TRIM32 polyubiquitinates MOV10 upon bicuculline stimulation and Trim32 knockdown prevents bicuculline-induced MOV10 degradation *via* proteasome.

4) Chronic hyperactivity promotes miRISC remodeling by causing the relative enrichment of Trim32 and depletion of MOV10 without affecting the level of Argonaute and Dicer – all members of the silencing complex.

5) Synaptic downscaling requires the differential distribution of translation regulators, proteasome and components of the miRISC in polysome, specifically the enrichment of Trim32 and depletion of MOV10 from high density fractions.

6) The direct interaction between regulators of translation and the proteasome is RNA dependent.

7) mTORC1-dependent Trim32 synthesis specifically happens via the activation of the p70 S6K pathway, to effectuate downscaling.

8) MOV10 degradation-dependent miRISC remodeling is a prerequisite for Arc expression which regulates sAMPAR distribution during synaptic downscaling.

9) mTORC1 driven synaptic scaling occurs through the 3’ UTR mediated post-transcriptional regulation of Arc rather than hyperactivity-induced transcription.

We have addressed all concerns of the reviewers and below are our detailed responses to each comment. All the reviews are very helpful and constructive. The manuscript has been amended to reflect the comments from reviewers. We believe that addition of these new data sets to existing line of evidences significantly improves the quality and strength of the manuscript.

Reviewer #1:

The manuscript by Srinivasan and colleagues describes a study addressing protein translation/degradation mechanisms which are relevant for homeostatic synaptic scaling. The authors describe that concomitant inhibition of the proteasome and translation blocks synaptic downscaling upon prolonged neuronal hyperactivity, and that bicuculline treatment triggers the degradation of MOV10 (dependent on proteasome activity) and upregulation of Trim32 expression (dependent on translation). Simultaneous blockade of proteasome activity and translation blocks bicuculline-induced changes in the expression levels of both MOV10 and Trim32. Since MOV10 levels are upregulated upon Trim32 silencing in neurons, the authors conclude that Trim32 (an E3 ligase) directly promotes MOV10 degradation. They also observe that MOV10 depletion decreases surface AMPAR levels and the amplitude of AMPAR-mediated mEPSCs, suggesting that it could be mechanistically involved in bicuculline-triggered downscaling, eventually through the regulation of Arc expression. This is an interesting study, which addresses the compelling idea that translation-dependent degradation of molecular targets mediates synaptic downscaling.

Authors:

We thank the reviewer for careful attention to the merits of the manuscript.

Reviewer:

The following aspects need to be addressed before publication is considered:

Comment # 1:

One initial evidence triggering the focus on MOV10 and Trim32 is their presence (along with proteasome subunits and translation factors) in polysomes isolated from hippocampal tissue. All these components are associated with polysomes under baseline conditions. Does prolonged neuronal activity, induced by bicuculline treatment, affect this association?

Authors:

We thank the reviewer for prompting us to examine the association of MOV10 and Trim32 with polysome upon bicuculline-induced hyperactivity. To address the activity-regulated association of MOV10 and Trim32 in polysome, we have analyzed the distribution of proteasome subunits, translation regulators and miRISC members in polysome fractions obtained from bicuculline-treated neurons. We have used primary cortical neurons in culture to obtain sufficient amount of samples for

polysome fractionation. These neurons were treated with 10µM bicuculline for 24 hours prior to fractionation. We observed a relative enrichment of 20S core subunits, p70 S6 kinase and its phosphorylated form and phosphorylated S6 in polysome fractions whereas Argonaute was depleted from polysome fractions obtained from bicuculline-induced neurons. Quantitative analysis of polysome association with MOV10 and Trim32 (normalized by polysome area under the curve) revealed that the prolonged neuronal activity led to depletion of MOV10 from polysome whereas abundance of Trim32 in polysome was enhanced (Figure 5).

Reviewer:

Comment #2. One central idea in interpreting the results in the paper is that Trim32 upregulation upon hyperactivity leads to MOV10 degradation, which is important for the downscaling process. Is MOV10 polyubiquitintion enhanced in bicuculline treated neurons?

Authors:

We agree that the reviewer identified an important missing piece in our experiments and we performed the suggested experiment. Hippocampal neurons were transduced with lentivirus expressing shRNA against Trim32 (previously characterized for effective knock-down) or control shRNA. Following effective knockdown of Trim32, these neurons were stimulated with bicuculline (10µM) in presence of proteasome inhibitor lactacystin (10µM) for 24 hours. Lactacystin was used to increase the pool of polyubiquitinated proteins. MOV10 containing protein complex was immunoprecipitated and its polyubiquitination was evaluated by western blot analysis using an antibody that can specifically recognize polyubiquitin conjugates but not monoubiquitinated proteins. Our western blot analysis demonstrates that the bicuculline-treatment triggers polyubiquitionation of MOV10 that requires the E3 ligase activity of Trim32. We noticed a striking reduction of polyubiquitination in the absence of Trim32 (Figure 6F), indicating that the miRISC-associated E3 ligase Trim32 is sufficient for polyubiquitination of MOV10 (Figure 6F).

Reviewer:

Comment #3. Despite the fact that the authors show that MOV10 is regulated by Trim32 (since Trim32 silencing upregulates MOV10 levels), they do not test whether this is relevant in synaptic downscaling. It would be important to test whether manipulating Trim32 levels 1) affects AMPAR subunits surface levels and the amplitude of mEPSCs, 2) interferes with bicuculline-induced synaptic scaling, and 3) affects bicuculline-triggered downregulation of MOV10. These are important

experiments, as they will reveal whether the described mechanism indeed mediates the scaling process, or rather is occurring during hyperactivity but not necessarily being a main contributor to downscaling.

Authors:
We agree with the reviewer that the relevance of Trim32 in homeostatic scaling needs to be analyzed following manipulation of its expression. At the prompting of the reviewer, we have provided three lines of evidence to support our hypothesis that Trim32 function is indeed required for homeostatic scaling. These include:

(i) Surface expression of AMPAR subunits was measured from bicuculline or vehicle treated hippocampal neurons transduced with lentivirus expressing shRNA against Trim32 or control shRNA. We observed that bicuculline-induced reduction of surface GluA1/A2 was prevented by Trim32 knock-down (31.35 ± 6.65% increase, $p<0.04$ for sGluA1 and 88.99 ± 5.52% increase, $p<0.0005$ for sGluA2 as compared to bicuculline treated neuron) (Figure 9H and 9I).

(ii) Trim32 knockdown in bicuculline-treated neurons showed an increase in mEPSC amplitude (2.82 ± 0.23 pA increase, $p<0.0001$) as compared to neurons incubated with bicuculline alone (Figure 9B), suggesting that the loss of Trim32 partially occludes downscaling.

(iii) Trim32 knockdown prevented bicuculline-triggered degradation of MOV10 by proteasome (199.41 ± 0.69% protected, $p<0.0001$) (Figure 6D and 6E). In addition, we have detected a modest but significant increase of MOV10 protein level after the loss of Trim32 under basal condition (58.3 ± 3.09% increase, $p<0.0001$) (Figure 6D and E).

These three lines of evidence from patch-clamp recordings, surface expression of AMPARs and analysis of MOV10 degradation following Trim32 knockdown emphasize that the Trim32-MOV10 axis is indeed a key contributor to bicuculline-induced downscaling rather than the mere consequence of chronic hyperactivity.

Reviewer:
Comment #4. Similarly, the authors need to test the effect of MOV10 knock-down on bicuculline-induced synaptic scaling. Does it occlude a further effect of bicuculline?

Authors:
At the prompting of the reviewer, we have analyzed the effect of MOV10 knockdown on bicuculline-induced synaptic scaling. Our data showed that MOV10 knockdown in

bicuculline-treated neurons did not reduce mEPSC amplitude further as compared to bicuculline-induced neurons expressing control shRNA (Figure 8I). However, the overexpression of MOV10 in bicuculline-treated neurons led to a modest but significant increase in mEPSC amplitude (1.64 ± 0.14 pA increase, $p<0.0001$) as compared to neurons incubated with bicuculline (Figure 8L), suggesting that the overexpression of MOV10 partially occludes downscaling. The ectopically expressed MOV10 is suitable for activity-dependent degradation by 26S proteasome, but the enhanced level of MOV10 even after application of bicuculline (due to the magnitude of its overexpression) is sufficient to cause partial occlusion of downscaling.

Reviewer:

Comment #5. In the last figure authors show that MOV10 silencing upregulates Arc, a known regulator of AMPAR endocytosis and synaptic downscaling. They need to show how MOV10 silencing affects bicuculline-induced upregulation of Arc.

Authors:

We have adopted this suggestion and investigated a possible mechanism of bicuculline-induced upregulation of Arc involving MOV10 silencing. To address this aspect, we have explored the mechanism of mTORC1-mediated Arc expression *via* Trim32-MOV10 axis using the following approach:

1) We have used the Arc 3'UTR fused luciferase construct as a reporter to investigate whether loss of miRISC activity is necessary for Arc expression during bicuculline-induced downscaling. We assessed the reporter activity on application of rapamycin or post knockdown of MOV10, Trim32 and Dicer (all members of miRISC).

2) We analyzed the transcriptional control of Arc by qRT-PCR following application of bicuculline with or without rapamycin or MOV10 knockdown.

We observed that chronic hyperactivity enhanced the Arc 3'UTR mediated reporter activity (61.1 ± 3.24% increase, $p<0.0003$). Knockdown of Trim32 or application of rapamycin prevented the bicuculline-induced reporter activity (Figure 10E). Our reporter assay data also showed that loss of MOV10 or Dicer under basal conditions was sufficient to increase its activity (77.34 ± 7.78% increase, $p<0.002$ for MOV10 RNAi and 77.4 ± 8.31% increase, $p<0.002$ for Dicer RNAi) (Figure 10E – F). Furthermore, MOV10 knockdown did not enhance bicuculline-induced reporter activity further (Figure 10E). We presume that the resultant levels of MOV10 that remains after MOV10 RNAi sets the threshold point of reporter activity and chronic hyperactivity cannot override this set point.

Our qRT-PCR analysis demonstrated that neither chronic hyperactivity nor MOV10 knockdown affects the levels of Arc mRNA (Figure 10H and 10I). Comprehensively, these observations suggest that the mTORC1-mediated Arc expression during downscaling is regulated at the post-transcriptional level involving Trim32-MOV10 axis.

Reviewer:

Comment #6. The authors find that bicuculline-induced changes in Trim32 and MOV10 expression, as well as synaptic downscaling and Arc upregulation , require activation of mTORC1 (as they are blocked by rapamycin). How is this effect produced? Some exploration of this mechanism would strengthen the manuscript.

Authors:

Our initial submission demonstrated that mTORC1 acts upstream of the Trim32-MOV10 axis. We have added further data to delineate the mechanistic details of the process. mTORC1 activation regulates two core components of the translation initiation machinery: p70 ribosomal S6 kinase (p70 S6K) and the eIF4E binding protein 1/2 (4E-BP1/2). Phosphorylation status of these two factors influenced by mTORC1 is a key determinant of translation initiation. p70 S6K activity regulates translation initiation by phosphorylating S6 protein of the 40S ribosomal subunit whereas phosphorylation of 4E-BP2 (predominant form of 4E-BP in brain) inhibits its repressor activity to promote translation initiation.

We analyzed the role of these two downstream effectors of mTORC1 in the regulation of Trim32 translation and subsequent MOV10 degradation. We observed that chronic hyperactivity triggered rapamycin-sensitive phosphorylation of p70 S6K but not 4EBP2 (Figure 7 G – I). The impact of p70 S6K phosphorylation on Trim32 and MOV10 was examined by inhibiting p70 S6K activity using its selective inhibitor, LY2584702 Tosylate. The inhibition of p70 S6K activity blocked bicuculline-induced Trim32 translation and consequent degradation of MOV10 (Figure 7 J – L). This observation mimics the inhibition of mTORC1 by rapamycin. The application of rapamycin also occluded bicuculline-induced downscaling (Figure 7A – C). Taken together, our data demonstrate that mTORC1-mediated regulation of the Trim32-MOV10 axis occurs through p70 S6K activity during downscaling.

We have investigated how mTORC1 influences the Trim32-MOV10 axis to regulate Arc expression during downscaling. We observed that the chronic hyperactivity enhanced the expression of Arc (Figure 10C – D). This enhancement of Arc expression (including Arc-3’UTR reporter activity and Arc protein level) was blocked

by rapamycin (Figure 10 C-D and 10G). Arc mRNA levels were not affected by chronic hyperactivity (Figure 10I). Notably, the bicuculline-induced reporter activity was prevented by Trim32 knockdown whereas loss of MOV10 alone is sufficient for enhancing the reporter expression (Figure 10 E). These data shows that mTORC1 –mediated regulation of Arc expression involves a post-transcriptional mechanism rather than a transcriptional change.

Reviewer:

Comment #7. English language needs to be revised in the manuscript.

Authors:

We thank the reviewer for giving the paper such careful attention. We have revised the English language in the entire manuscript for better clarity of data presented. We hope that the language revision will enhance the quality of the manuscript for readers.

Reviewer #2:

This manuscript investigated how proteasome (UPS degradation) and ribosome (translation) work coordinately to control homeostatic plasticity of excitatory synapses. Several studies have reported the effect of a translation inhibitor (anisomycin, cycloheximide, rapamycin…) or a proteasome inhibitor (lactacystin or a less-specific MG132) in synaptic scaling phenomenon. However, to my knowledge, this is the first study to examine both kinds of inhibitors, alone and in combination, in synaptic scaling in cultured neurons. The authors proposed a molecular model that translation of Trim32 (an E3 ligase) mediates proteasome-dependent MOV10 degradation. MOV10 reduction upregulates Arc synthesis, leading to decreased surface AMPARs. Despite an interesting model, many conclusions, from the title to several subheadings, are overstated to hamper my enthusiasm to support its publication.

Authors:

We appreciate the reviewer's careful attention to the merits of the manuscript and insightful comments. We have now added several lines of evidence to justify our model that mTORC1 –mediated Trim32 translation with concomitant degradation of MOV10 underlies miRISC remodeling during synaptic downscaling. We strongly believe that the reviewer's insightful comments immensely helped us to strengthen the manuscript with appropriate experiments. We have provided a detailed justification against each concern that is mentioned below.

Reviewer:

Comment # 1**:** The title "Homeostatic scaling is driven by a translation-dependent degradation axis that recruit miRISC remodeling" appears to overstate the findings from the study. The authors suggested that translation of Trim32 is a prerequisite to invoke proteasome-mediated degradation of MOV10 and consequently affect miRISC remodeling to drive synaptic scaling. However, there is no evidence to show that the TRIM32-MOV10-miRISC axis is sufficient to drive synaptic scaling.

Authors:

We thank the reviewer for prompting us to examine the necessity of the Trim32-MOV10-miRISC axis in synaptic scaling. To address the importance of this tripartite complex in downscaling, we have provided following lines of evidences:

1) Loss of Trim32 or overexpression of MOV10 partially impairs bicuculline-induced downscaling (Figure 8 – 9).
2) Bicuculline-induced reduction of surface AMPARs is prevented by Trim32 knockdown (Figure 9).
3) Loss of MOV10, that mimics bicuculline-induced downscaling, is sufficient to reduce sAMPARs (Figure 8).
4) Chronic hyperactivity changes the composition of miRISC (known as miRISC remodeling) by promoting the enrichment of Trim32 with concomitant depletion of MOV10 from the complex without affecting the other members, such as Argonaute and Dicer (Figure 6).

We have provided a detailed explanation for each of our observations in the manuscript as well as in the rebuttal letter to resolve the apparent discrepancy between the title of the manuscript and the focus of this study. We believe that additional data provided in the manuscript will convince the readers that the manuscript title and subheadings are in sync with the overall concept and data presented.

Reviewer:

Although the protein levels of TRIM32 and MOV10 showed a good opposite correlation to support the authors' hypothetic model about synaptic scaling in the presence of anisomycin/ rapamycin, lactacystin or both, they did not provide data to show that TRIM32 ubiquitinates MOV10 and the disruption of TRIM32-mediated MOV10 degradation is sufficient to abolish synaptic scaling.

Authors:

Reviewer #1 raised a similar concern and this issue is extensively discussed in our response to reviewer #1, comment #2 and # 3. The detailed explanation on this valuable point has been included in the revised manuscript. We have provided the following lines of evidence.

(i) We have analyzed the Trim32-mediated degradation of MOV10 by Ubiquitin Proteasome System (UPS) upon chronic hyperactivity. Our western blot analysis showed that the loss of Trim32 prevented bicuculline-induced degradation of MOV10 (199.41 ± 0.69% protected, $p<0.0001$) (Figure 6D – E). Moreover, Trim32 knockdown enhanced MOV10 expression (58.3 ± 3.09% increase, $p<0.0001$) under basal conditions (Figure 6D-E).

(ii) We examined whether Trim32 activity is sufficient for bicuculline-induced MOV10 degradation by proteasome. MOV10 was immunoprecipitated from bicuculline-treated neurons in presence or absence of Trim32. Polyubiquitination of immunoprecipitated MOV10 was analyzed by an antibody that can specifically recognize polyubiquitin conjugates (FK1 from Enzo Lifesciences). These neurons were incubated with lactacystin (a specific proteasome inhibitor) to enhance the pool of polyubiquitinated proteins. We observed that chronic hyperactivity triggered polyubiquitination of MOV10. This bicuculline-induced polyubiquitination of MOV10 was prevented by Trim32 knockdown (Figure 6F).

(iii) To establish that Trim32 –mediated MOV10 degradation is sufficient for synaptic scaling, we measured mEPSCs from bicuculline-treated neurons following Trim32 RNAi. Hyperactivity-induced downscaling was partially occluded by Trim32 knockdown in bicuculline-treated neurons (2.82 ± 0.23 pA increase as compared to bicuculline-treated neurons expressing control shRNA, $p<0.0001$) (Figure 9A – B). Overexpression of MOV10 also enhanced the mEPSC amplitude (1.64 ± 0.14 pA increase as compared to bicuculline-treated neurons, $p<0.0001$) following hyperactivity (Figure 8 K-L). Although ectopically expressed MOV10 is suitable for UPS-mediated degradation, we believe that sustained enhancement of the overexpressed MOV10 levels even after bicuculline treatment is sufficient for the modest but significant increase in mEPSC amplitude. Our data demonstrates that scaling is partially impaired when Trim32 and MOV10 levels are reversed.

Reviewer:

Moreover, to my knowledge, MOV10 and TRIM32 do not function exclusively only in the miRISC machinery, so the authors need to address what "miRISC remodeling" means. Does the silencing activity of miRISC change by the bicuculline treatment?

Authors:

We agree with the reviewer that there are reports showing that Trim32 and MOV10 individually function outside the miRISC. However, we would like to highlight the extensive literature demonstrating that Trim32 and MOV10 function as integral components of the miRISC and their individual activity modulates miRISC function (Davis et al., 2008; Banerjee et al., 2009; Schwamborn et al., 2009).

According to existing literature (Schratt, 2009); compositional changes within the miRISC is termed as 'miRISC remodeling'. Such compositional changes in the silencing complex may alter its activity. Hence we have used the term "remodeling" to denote alterations in the composition and function of miRISC during downscaling. Based on our biochemical evidences, we believe that the miRISC remodeling is achieved through Trim32 translation-dependent degradation of MOV10 by proteasome (Figure 6). However, we agree with the reviewer that more experiments were needed in our initial submission to justify the term. We have provided the following lines of evidence.

(i) We immunoprecipitated Argonaute-containing protein complex from bicuculline-induced neurons as existing literature showed that affinity-purified Argonaute complex is essentially enriched with miRISC factors (Meister et al., 2005). We find Trim32 and MOV10 to be associated with the Dicer and Argonaute-containing miRISC. We find that chronic hyperactivity promotes the enrichment of Trim32 and depletion of MOV10 within the Argonaute-containing miRISC. However, hyperactivity does not affect Argonaute and Dicer, suggesting that specific factors are targeted for a compositional change in the miRISC upon bicuculline treatment (Figure 6G). Hence, we infer that compositional changes in the miRISC are happening due to Trim32 and MOV10, at-least in the context of synaptic downscaling.

(ii) We have investigated the impact of chronic bicuculline treatment on the silencing activity of the miRISC following knockdown of miRISC factors – Trim32, MOV10 and Dicer. The reporter assay engages the 3'UTR of Arc mRNA to drive reporter expression. We have focused onto the Arc 3'UTR for the following reasons:

(a) Multiple miRNAs binding site present in the 3'UTR of Arc is indicative of its association with miRISC.
(b) The 3'UTR of Arc has been shown to regulate post-transcriptional control of gene expression in hippocampal neurons.
(c) Knockdown of miRISC member MOV10 enhances the expression of Arc protein.

We observed that the Arc 3'UTR –mediated reporter expression was enhanced (61.1 ± 3.24% increase, p<0.0003) by chronic application of bicuculline. This enhancement of reporter expression upon chronic hyperactivity was prevented by Trim32 knockdown (Figure 10E). Loss of MOV10, that mimics biochemical events during scaling, was sufficient to increase (77.34 ± 7.78% increase, p<0.002) the reporter expression under basal condition. Similar to MOV10 RNAi, Dicer knockdown also enhanced the reporter expression (77.4 ± 8.31 % increase, p<0.002) (Figure 10F).

Reviewer:
From their data, I agree that the TRIM32-MOV10-Arc axis may contribute to synaptic scaling but find no evidence to support the sufficiency of this axis to drive synaptic scaling.

Authors:

To ascertain the contribution of Trim32-MOV10-Arc axis in synaptic scaling, we provided two lines evidences. These include:

(i) Loss of Trim32 or overexpression of MOV10 partially impairs bicuculline-induced downscaling (Figure 8–9). This observation is based on our electrophysiology data showing that the knockdown of Trim32 or ectopic expression of MOV10 in bicuculline-induced neurons enhanced mEPSC amplitude as compared to neuron incubated with bicuculline (2.82 ± 0.23 pA increase, p<0.0001 for Trim32 knockdown; 1.64 ± 0.14 pA increase, p<0.0001 for MOV10 overexpression) (Figure 9B and 8L).

(ii) MOV10 RNAi or bicuculline-treatment enhanced the expression of Arc protein in a similar manner (113.1 ± 15.7% increase, p<0.002 for MOV10 shRNA # 1 and 173.8 ± 7.45% increase, p<0.0001 for MOV10 shRNA # 2; 132.1 ± 27.45% increase, p<0.04 for bicuculline-treatment) (Figure 10A-D). Arc 3'UTR –mediated reporter expression was enhanced (61.1 ± 3.24% increase, p<0.0003) upon hyperactivity (Figure 10E) which was prevented by Trim32 knockdown (Figure 10E). We noticed an enhancement of reporter activity (77.34 ± 7.78% increase, p<0.002) upon loss of MOV10 under basal conditions (Figure 10E), which resembled bicuculline-induced enhancement of reporter activity. As MOV10 RNAi mimics bicuculline-induced degradation of

MOV10, similar enhancement of Arc reporter activity implies that MOV10 degradation alone is sufficient to alter miRISC function and concomitant Arc expression during scaling.

(iii) Arc has been shown to regulate endocytosis of AMPARs to effectuate downscaling. We observed that bicuculline-induced reduction in sAMPARs was prevented by the Trim32 RNAi (31.35 ± 6.65% increase, $p<0.04$ for sGluA1; 88.99 ± 5.52% increase for sGluA2, $p<0.0005$ as compared to sGluA1/A2 level detected after bicuculline treatment) (Figure 9D - I). MOV10 RNAi, on the other hand, reduced sGluA1/A2 distribution (35.03 ± 9.35 % reduction, $p<0.01$ for sGluA1; 49.4 ± 12.9% reduction for sGluA2, $p<0.01$) under basal conditions (Figure 8A – D); in a manner similar to that observed during chronic bicuculline treatment. Comprehensively, these data sets indeed emphasize that the Trim32-MOV10-Arc axis is necessary for synaptic downscaling.

Reviewer:

Comment #2: Figure 3 and the subheading "RNA-dependent tethering of the proteasome and translation regulators". The RNase treatment digests RNA to make polyribosomes to 80S monosomes. If proteasomes interact with ribosomes in an RNA-independent manner, one could also observe the reduction of proteasome subunits in the heavy density of sucrose gradient. What the authors need to do is to perform co-immunoprecipitation (co-IP) by using polysomal fraction treated with or without RNase.

Authors:

We thank the reviewer for careful attention to our manuscript. We agree with the reviewer that empirical evidence for an RNA-dependent interaction between polysome-associated ribosomes and proteasomes is necessary to support our claim. As suggested by the reviewer, we have performed co-immunoprecipitation experiments from polysomal fractions treated with or without RNase. We used a transgenic mouse expressing HA-tagged ribosomal protein Rpl22, based on our previous polysomal analysis showing that HA-Rpl22 containing ribosomes are distributed throughout the heavy sucrose gradient fractions (Figure 4C-D). We argue that affinity purification of HA-Rpl22 from these heavy sucrose fractions would essentially co-precipitate polysome- associated protein complexes. Cytoplasmic lysate from hippocampus was treated with or without RNase prior to density gradient fractionation. Heavy sucrose gradient fractions were pooled and used for affinity purification using an antibody against HA. Western blot analysis from HA-Affinity purified protein complex detected the presence of 20S proteasome core subunits and 19S cap subunits (Rpt1 and Rpt6) along with translation regulators, such as eIF4E and p70 S6K in heavy density polysome fractions. We observed that

this association was abolished by RNase treatment (Figure 4J). Therefore, our data endorses an RNA-dependent interaction between proteasomes and ribosomes engaged in active translation.

Reviewer:

Moreover, it should be able to run all isolated sucrose fractions in a single 15-well gel for immunoblotting, so any specific reason not to do so?

Authors:

We understand the concern of the reviewer that the running a single 15-well gel for immunoblotting is preferable to compare the variations in each fraction. Unfortunately, we do not have a 15-well gel apparatus for immunoblotting. We have marked the beginning and end of the lanes in each blot represented in all photomicrograph representing polysome analysis.

Reviewer:

Comment #3: Figure 4, "Interaction between proteasome and actively translating RNA-associated polyribosomes." Here, the authors simply used several co-IPs to reach the conclusion. They also cited a reference (Krichevsky et al, 2003) to claim that the interaction of MOV10 with ribosomes is crucial as it gives credence to the association of miRNAs with polysomes, as per previous reports (Krichevsky et al, 2003). However, I found no evidence in this paper or other literatures to support that "polysome-associated MOV10" is equivalent to "the association of miRNAs with polysomes". After all, MOV10 is an RNA helicase, which binds to and unwinds RNA secondary structure to facilitate posttranscriptional regulation, so its association with polysomes is not surprising.

Authors:

We have provided the following lines of evidence to support our claim that the association of MOV10 with polysome is equivalent of miRISC interaction with actively translating transcripts:

(i) Immunoprecipitation analysis of Argonaute-containing protein complex also co-precipitated Dicer, MOV10 and Trim32. Bicuculline-induced chronic hyperactivity led to an enrichment of Trim32 and depletion of MOV10 within the silencing complex. This observation is based on western blot analysis of immunoprecipitated protein complex containing Dicer and Argonaute that was obtained from bicuculline-treated neurons (Figure 6G).

(ii) Western blot analysis of polysome fractions obtained from bicuculline or vehicle treated neurons shows that chronic hyperactivity promotes depletion of MOV10 as well as Argonaute and enhances Trim32 abundance in polysome (Figure 5).

(iii) MOV10 RNAi is sufficient to reverse the miRISC-mediated post-transcriptional silencing of Arc expression (please see response to comment #1).

Existing literature demonstrates that miRNAs along with components of the miRISC, such as Argonaute and MOV10 are associated with polysome (Krichevsky et al., 2003; Nottrott et al., 2006; Kenny et al., 2014). We agree with the reviewer that MOV10 is a RNA helicase and its presence in the polysome may be intuitive, but the bicuculline-dependent dynamic association of MOV10, and Argonaute in polysomes, the observed physical interactions between MOV10, Ago and Dicer, and the ability of MOV10 to influence the silencing activity of miRISC allow us to infer that the presence of MOV10 in polysome fractions is equivalent of polysome association with miRISC at least in the context of bicuculline-induced downscaling.

Reviewer:
Comment #4: Figure 5, "Protein synthesis drives mTORC1-dependent proteasomal degradation to cause miRISC remodelling during synaptic downscaling". The authors used the change of TRIM32 and MOV10 expression to indicate miRISC remodeling but provided no empirical evidence to show altered miRISC activity.

Authors:
We agree that the reviewer identified an important missing piece in our experiment and we have analyzed the regulation of miRISC activity by the Trim32-MOV10 axis. Our observations are as follows:

a) Immunoprecipitation analysis demonstrated that Trim32 and MOV10 are integral components of the Argonaute and Dicer-containing miRISC. Chronic hyperactivity remodeled the miRISC by inducing the enrichment of Trim32 and concomitant depletion of MOV10 from the silencing complex, while keeping Dicer unaltered (Figure 6G).

b) After identifying compositional changes within the miRISC, we used an Arc 3’ UTR based reporter to measure the miRISC functionality during bicuculline induced scaling. All the results have been discussed in detail in response to comment #1 of the reviewer. In brief, bicuculline-mediated reversal in the silencing activity of miRISC (measured as the enhancement of Arc-luciferase reporter activity) was

abrogated by Trim32 knockdown; whereas MOV10 RNAi was sufficient to cause miRISC silencing even in basal conditions (Figure 10).

Comprehensively, the reporter assay data shows that the bicuculline-induced miRISC remodeling causes alteration in miRISC activity.

Reviewer:

Moreover, how does "protein synthesis drives mTORC1-depednent proteasomal degradation" just based on the presence of anisomycin or rapamycin? If so, why not conclude mTORC1-dependent autophagosomal degradation?

Authors:

The degradation of proteins occurs by both Ubiquitin Proteasome System (UPS) and autophagy. Proteasome-mediated degradation of cellular proteins exhibits a high degree of specificity by involving selective enzymatic cascade of reactions that polyubiquitinates proteins. The polyubiquitination of proteins is regulated by a specific E3 ligase. Protein degradation by autophagy primarily involves lysosomes (Pohl and Dikic, 2019). Although, autophagosomal degradation can occur by both ubiquitin dependent and independent process, the UPS-dependent degradation predominantly occurs by polyubiquitination *via* enzymatic cascade. UPS -mediated degradation of proteins is lactacystin sensitive, whereas protein degradation by autophagy is not.

We observed that the chronic hyperactivity polyubiquitinates MOV10 exclusively by the E3 ligase Trim32. Our data showed that the bicuculline-induced degradation of MOV10 by proteasome is blocked by knockdown of Trim32 or lactacystin treatment (Figure 6 – 7). Taken together, these observations suggest that the Trim32 –mediated MOV10 degradation indeed occurs through UPS rather than involving autophagosome.

Reviewer:

Comment #5: The authors showed that MOV10-KD reduced surface AMPARs. How about treating MOV10-KD neurons with bicuculline? Could hyperexcitability promote synaptic scaling in MOV10-KD neurons or such a scaling is completely abolished? How about TRIM32-KD or overexpression? If the model is correct, the synaptic scaling would be abolished or partially impaired in the absence of TRIM32.

Authors:

A similar concern was raised by reviewer # 1 and this is extensively discussed in our response to comments # 3 and # 4. As suggested by the reviewers, we have

measured mEPSCs from bicuculline-induced neurons following MOV10 kockdown. We observed that hyperactivity-induced reduction in mEPSC amplitude was not altered by the loss of MOV10 (Figure 8I). We presume that MOV10 knockdown set the threshold for synaptic strength and application of bicuculline cannot further override this set point.

To delineate the role of MOV10 in synaptic scaling upon hyperactivity, we have measured mEPSCs from bicuculline-induced neurons overexpressing MOV10. We observed that MOV10 overexpression caused a modest but significant increase in mEPSC amplitude (1.64 ± 0.14 pA increase, $p<0.0001$) following bicuculline-treatment (Figure 8L). As suggested by the reviewer, the effect Trim32 knockdown on downscaling was also analyzed by measuring mEPSC from bicuculline-induced neurons post Trim32 RNAi. We observed that loss of Trim32 in bicuculline-induced neurons increased the mEPSC amplitude (2.82 ± 0.23 pA increase, $p<0.0001$) as compared to neurons expressing control shRNA under similar conditions (Figure 9B).

Comprehensively, our data suggests that the reversal of bicuculline-regulated Trim32 and MOV10 level by genetic manipulation partially occludes downscaling.

Reviewer:

Comment #6: The data in Figure 7A let me question the KD efficiency of MOV10. The shRNA#1 did not reduce MOV10 level yet it promoted Arc expression (Figure 7B). As Arc is an immediately early gene sensitive to neuronal activation, it is not surprising that bicuculline increases Arc expression, which could be blocked by rapamycin (Figure 7C, D). This phenomenon could be caused by transcriptional upregulation of Arc instead of miRISC-mediated posttranscriptional regulation of Arc. Simple experiments like monitoring Arc mRNA and protein levels in the presence of transcription inhibitor or rapamycin can answer this question.

Authors:

We thank the reviewer for a careful attention to the data presented in Figure 7A – 7B (represented in the initial submission). The photomicrograph showing apparent discrepancy between MOV10 knockdown by shRNA # 1 and Arc expression can be resolved by normalizing with Tuj. We would like to highlight that the Tuj1 level was higher in lane # 2 (MOV10 knock-down by shRNA#1) as compared to lane # 1 (no knock-down by control shRNA) in the photomicrograph. Normalization with Tuj1 indicated a knockdown of MOV10 to a lesser extent (as per photomicrograph represented in the initial submission). This observation is consistent with the degree of knockdown efficacy between shRNA# 1 and shRNA # 2 against MOV10. However, we have repeated this experiment and added a revised blot and quantitation in the

Figure 10A-B for better clarity to show that the MOV10 knockdown led to an increase (113.1 ± 15.7% increase, p<0.002 for shRNA # 1 and 173.8 ± 7.45% increase, p<0.0001 for shRNA # 2) (Figure 10A-B) in Arc expression.

We have examined the mechanism of mTORC1–mediated regulation of Arc expression by following approaches:

1) Measured the mRNA and protein level of Arc upon bicuculline-treatment in presence or absence of rapamycin.

2) Analyzed the bicuculline-induced reporter expression –mediated by Arc 3'UTR. The reporter assay was performed following inhibition of mTORC1 by rapamycin and also after knockdown of miRISC members; MOV10, Trim32 and Dicer.

Quantitative PCR (qPCR) analysis of Arc mRNA from bicuculline-induced neurons in presence or absence of rapamycin did not show any change in the transcript level (Figure 10I). We also observed that MOV10 knockdown did not influence the Arc mRNA (Figure 10H).

In contrast, our western blot analysis showed that hyperactivity led to an increase (132.1 ± 27.45% increase, p<0.04) in Arc protein level (Figure 10C-D), that was abrogated by rapamycin (Figure 10C-D). Similar to hyperactivity-induced enhanced level of Arc protein, loss of MOV10 also increased the (113.1 ± 15.7% increase, p<0.002 for shRNA # 1 and 173.8 ± 7.45% increase, p<0.0001 for shRNA # 2) Arc protein (Figure 10A-B). Comparative analysis of transcript and protein level of Arc following application of bicuculline or loss of MOV10 rules out hyperactivity-induced transcriptional change in Arc expression and indicates a post-transcriptional mechanism.

Our reporter assay data showed that the hyperactivity enhanced the Arc 3'UTR-mediated reporter activity (61.1 ± 3.24% increase, p<0.0003), indicating the occurrence of Arc translation. MOV10 knockdown was sufficient to enhance (77.34 ± 7.78% increase, p<0.002) the reporter activity under basal condition (Figure 10E). These observations provide an empirical evidence for bicuculline-induced post-transcriptional mechanism regulating Arc expression *via* 3'UTR of the transcript known to interact with miRISC.

Reviewer #3:

Homeostatic scaling is driven by a translation dependent degradation axis that recruits miRISC remodelling

Srinivasan et al.

This manuscript by Srinivasan et al. investigates the molecular mechanisms at play for the expression of the homeostatic form of down synaptic scaling. In the light of recent publications showing that both protein synthesis and degradation are recruited during this process the authors explore the possibility of a coupling of both machineries. Indeed, they found that elements of the ribosome could be co-immunoprecipitated with proteasomal proteins and vice versa. Interesting they were able to temporally resolve the cascade that leads to synaptic AMPAR removal. This cascade involves first the synthesis of TRIM32 that leads to miRISC remodelling via the degradation of MOV10 allowing consequently the synthesis of key proteins for the expression of synaptic scaling like Arc. To do so the authors used electro-physiological recordings, immunostainings, polysome profiling and traditional biochemistry. The study was rigorously conducted, the manuscript is well written, and the content is in my opinion of interest to the readership of PLOS Biology. I think this paper should be accepted for publication after the authors have discussed the minor concern.

Authors:

We thank the reviewer for appreciating the concept outlined in the manuscript and highlighting the strength of the manuscript for publication in PLoS Biology. We have now added several additional data in the revised manuscript to strengthen our claim that the Trim32 translation-dependent degradation of MOV10 by proteasome remodels miRISC to drive synaptic downscaling. We have shown that the miRISC remodelling regulates Arc expression *via* post-transcriptional mechanism to regulate synaptic AMPARs. We have addressed all minor concerns raised below to make the manuscript more succinct.

Reviewer:

Minor concerns:

Comment #1: Figure 3B: It looks like MOV10 and TRIM32 are excluded from faction 8/11/15. Is this a real result? Or is it a technical issue? The authors should discuss this observation in the manuscript.

Authors:

We thank the reviewer for careful attention to our manuscript. The exclusion of MOV10 and Trim32 in fractions 8/11/15 are due to technical issues. The absence of

Trim32 and MOV10 in these fractions is possibly due to loss of protein precipitate during the TCA precipitation step. We have discussed this point in the result section of the manuscript. We have investigated the polysome association of MOV10 and Trim32 following bicuculline treatment. Western blot analysis of this experiment showed that both Trim32 and MOV10 were distributed throughout the high density sucrose fractions containing polysome. The quantitative analysis showed that the bicuculline-induced hyperactivity led to enhanced Trim32 abundance and depletion of MOV10 from polysome (Figure 5).

Reviewer:
Comment #2: miRISC should be spelt out in the introduction: microRNA induced silencing complex.

Authors:
We have corrected this omission in introduction.

Reviewer:
Comment #3: Figure 2: it might be nicer to have E and F at the bottom of the figure.

Authors:
We thank the reviewer for the suggestion. We have rearranged the Figure 2 as suggested by the reviewer.

Reviewer:
Comment #4: Figure 4B: the drawing is misleading. Could the authors draw a ribosome with two subunits and the big one carrying an HA-tag?

Authors:
We thank the reviewer for pointing out this discrepancy. We have revised the drawing as suggested by the reviewer to make this figure discernible.

Reviewer:
Comment #5: typo line 559: hippocamapal => hippocampal

Authors:
We have corrected this mistake.

We believe that the additional data presented in the revised manuscript has significantly enhanced the quality and strength of the study. I would greatly appreciate if you consider the revised version of the manuscript for peer review and

publication in PLoS Biology. We apologize for the long delay in communicating the revised manuscript due to the unforeseeable second wave of the pandemic in India.

Warm regards,

Sourav Banerjee

References:

Banerjee S, Neveu P, Kosik KS (2009) A Coordinated Local Translational Control Point at the Synapse Involving Relief from Silencing and MOV10 Degradation. Neuron 64:871–884.

Davis TH, Cuellar TL, Koch SM, Barker AJ, Harfe BD, McManus MT, Ullian EM (2008) Conditional loss of dicer disrupts cellular and tissue morphogenesis in the cortex and hippocampus. J Neurosci 28:4322–4330.

Kenny PJ, Zhou H, Kim M, Skariah G, Khetani RS, Drnevich J, Arcila ML, Kosik KS, Ceman S (2014) MOV10 and FMRP Regulate AGO2 Association with MicroRNA Recognition Elements. Cell Rep 9:1729–1741.

Krichevsky AM, King KS, Donahue CP, Khrapko K, Kosik KS (2003) A microRNA array reveals extensive regulation of microRNAs during brain development. Rna 9:1274–1281.

Meister G, Landthaler M, Peters L, Chen PY, Urlaub H, Lührmann R, Tuschl T (2005) Identification of novel argonaute-associated proteins. Curr Biol 15:2149–2155.

Nottrott S, Simard MJ, Richter JD (2006) Human let-7a miRNA blocks protein production on actively translating polyribosomes. Nat Struct Mol Biol 13:1108–1114.

Pohl C, Dikic I (2019) Cellular quality control by the ubiquitin-proteasome system and autophagy. Science (80-) 366:818–822.

Schratt G (2009) MicroRNAs at the synapse. Nat Rev Neurosci 10:842–849.

Schwamborn JC, Berezikov E, Knoblich JA (2009) The TRIM-NHL Protein TRIM32 Activates MicroRNAs and Prevents Self-Renewal in Mouse Neural Progenitors. Cell 136:913–925.
